# Rapid, Unbiased PRRSV Strain Detection Using MinION Direct RNA Sequencing and Bioinformatics Tools

**DOI:** 10.3390/v11121132

**Published:** 2019-12-07

**Authors:** Shaoyuan Tan, Cheryl M.T. Dvorak, Michael P. Murtaugh

**Affiliations:** Department of Veterinary and Biomedical Sciences, College of Veterinary Medicine, University of Minnesota, St. Paul, MN 55108, USA; tanxx606@umn.edu (S.T.); murta001@umn.edu (M.P.M.)

**Keywords:** MinION, direct RNA sequencing, PRRSV, whole genome sequencing, analytical sensitivity, strain level detection, quantitation, co-infection, bioinformatics

## Abstract

Prompt detection and effective control of porcine reproductive and respiratory syndrome virus (PRRSV) during outbreaks is important given its immense adverse impact on the swine industry. However, the diagnostic process can be challenging due to the high genetic diversity and high mutation rate of PRRSV. A diagnostic method that can provide more detailed genetic information about pathogens is urgently needed. In this study, we evaluated the ability of Oxford Nanopore MinION direct RNA sequencing to generate a PRRSV whole genome sequence and detect and discriminate virus at the strain-level. A nearly full length PRRSV genome was successfully generated from raw sequence reads, achieving an accuracy of 96% after consensus genome generation. Direct RNA sequencing reliably detected the PRRSV strain present with an accuracy of 99.9% using as few as 5 raw sequencing reads and successfully differentiated multiple co-infecting strains present in a sample. In addition, PRRSV strain information was obtained from clinical samples containing 10^4^ to 10^6^ viral copies or more within 6 hours of sequencing. Overall, direct viral RNA sequencing followed by bioinformatic analysis proves to be a promising approach for identification of the viral strain or strains involved in clinical infections, allowing for more precise prevention and control strategies during PRRSV outbreaks.

## 1. Introduction

The swine industry plays an important role in feeding the world, as pork is one of the highest consumed animal proteins in the world [1,2]. Emerging and re-emerging viral infectious diseases have been posing great challenges to the swine industry, among which porcine reproductive and respiratory syndrome (PRRS) is one of the most devastating diseases [3,4]. PRRS virus (PRRSV) is the causative agent of PRRS which contains a positive-sense, single-stranded, polyadenylated, 15 kb RNA genome [5]. PRRSV is categorized into two genotypes, type 1 (European type) and type 2 (North American type), which differ by approximately 40% at the genomic level between the two genotypes [6,7,8], and strains within each genotype also vary considerably with genomic differences as high as 20% [9].

Globally, PRRS remains a threat to the swine industry despite many years of combined efforts to combat and control infection and disease [10]. One of the challenges for PRRSV control is the frequent recurrence of PRRS outbreaks in swine farms [11], with a prediction that of farms reporting an outbreak today, 71% will have a recurrence of PRRSV infection within the following two years [12]. The PRRS recurrence is either caused by introduction of a new strain or the resident virus strain. The knowledge of which type of PRRS recurrence is crucial to determine the necessary control methods. Controlling against a new introduction from outside the farm indicates a need for enhanced biosecurity, while a re-break of a resident strain suggests better strategies for an elimination or vaccination program are needed. Another big challenge for PRRS control is that PRRSV vaccine is not completely effective at preventing and controlling infection due to the high genetic diversity of the virus, thus outbreaks still occur in vaccinated herds [13,14,15,16]. A diagnostic method which can provide genetic information about the strain causing infection would allow for identification of potential reasons for vaccination failure, such as limited cross-protection due to high genetic divergence from vaccine [17], or in the case of genetic similarity to vaccine, perhaps a reversion to virulence (escape mutation) of the vaccine itself [18]. In addition to the clinical challenges mentioned above, PRRSV has widely divergent genetic lineages and is a rapidly evolving pathogen with novel variants which seem to be more divergent and virulent than those in the past [10,19,20,21]. The continuous emergence of new virulent strains causes unexpected devastating outbreaks, such as the severe outbreaks of HP-PRRSV in China and MN184 and NADC30 outbreaks in the United States [22,23,24,25]. The increasing incidence of co-infections of multiple strains further complicates PRRS diagnosis and control [26]. Hence, diagnostic tools that can provide more genetic information are extremely important for investigation, prevention, and control strategies for PRRSV outbreaks.

Prompt detection of pathogens during an outbreak is essential for efficient disease control. Real-time quantitative reverse transcription polymerase chain reaction (RT-qPCR) testing, which has the advantages of short turnaround time and high sensitivity [27,28], is currently the primary molecular diagnostic method for PRRSV detection and is performed routinely in diagnostic laboratories. The current PCR methods can quickly detect the presence of PRRSV in general [29], but cannot identify specific strains unless targeted primers are designed which requires prior knowledge about the strains of interest [26,30]. Nucleic acid sequencing technologies have been incorporated as a new diagnostic tool in recent years to provide necessary support, such as strain information, for PCR in clinical sample diagnostics [31,32,33]. Sequencing approaches have been applied successfully to various fields in virology so far, such as the discovery of novel viruses/strains, whole viral genome sequencing, quasispecies detection, and epidemiological investigations [34,35]. The fact that sequencing is robust and does not need prior knowledge of the pathogens/strains under detection is particularly important for rapid responses to highly variable pathogens, such as PRRSV [36]. The routine use of viral genome sequencing and genomic surveillance will not only serve as a powerful tool for PRRSV detection [37], but also provides researchers with a better understanding of PRRSV epidemiology and how the virus transmits, spreads, and evolves, thus facilitating effective prevention and control measures [38].

Conventional sequencing methods for RNA viruses usually includes reverse transcription and PCR amplification during library preparation which is then followed by amplicon sequencing. These extra steps not only introduce bias, but also increase the sequencing time, making rapid diagnosis difficult [39,40,41]. The Oxford Nanopore MinION sequencer allows for sequencing of the RNA molecule directly, in its native format. This feature, together with other characteristics such as low start-up costs, portability, and real-time data streaming, makes the Oxford Nanopore MinION sequencer a good candidate for rapid RNA virus detection, even in resource-limited or remote areas [40,42,43]. Influenza virus was the first pathogen to be successfully sequenced in its native RNA format by direct RNA sequencing (DRS) using Oxford Nanopore MinION technology [44]. Since then, studies have been performed for other viruses, confirming the potential of MinION technology to aid in the detection of infectious viral agents [45,46]. PRRSV whole genome sequencing (WGS) has been carried out previously using traditional Sanger sequencing [10] and next-generation short-read sequencing platforms [47]. Incorporating a bioinformatic approach, we systematically evaluated and standardized third-generation long-read MinION DRS for PRRSV whole genome generation and determined its ability to detect the viral strain present, its analytical sensitivity for strain-level diagnosis of clinical samples, and its feasibility for differentiation of co-existing multiple strains in a single sample. To our knowledge, our study is the first time PRRSV has been sequenced in its native RNA format without amplification. 

## 2. Materials and Methods

### 2.1. Viral Strains and Samples

A PRRSV type 2 isolate, VR2332 (GenBank: EF536003.1), was used as the main reference strain. A PRRSV type 1 isolate PRRSV1/USA/Lab6 (SDEU, GenBank: MN175678) and PRRSV type 2 isolate PRRSV2/USA/Lab3 (1-7-4, GenBank: MN175677) were used for experiments examining the detection of multiple viral isolates in a single sample. All viral isolates were propagated on MARC 145 cells as previously described [48]. Clinical samples and the corresponding ORF5 sequences from Sanger sequencing, were obtained from. Sunil Kumar Mor at the University of Minnesota Veterinary Diagnostic Laboratory (St. Paul, MN, USA).

### 2.2. RNA Extraction and Viral Copy Number Determination

PRRSV RNA was extracted from cell culture supernatants, virus-negative pig serum spiked with PRRSV, and clinical PRRSV-positive serum samples using the QIAamp Viral RNA mini kit (Qiagen, Germantown, MD) following manufacturer’s instructions without the addition of carrier RNA and with a final elution in 50 μL nuclease-free water. A high concentration PRRSV stock (supernatants from virus grown in MARC-145 cells) was extracted to generate a large amount of high concentration RNA for whole genome sequencing. Generation of known concentrations of the virus in serum samples (spike-in samples) was performed by adding the PRRSV stock to virus-negative pig serum, half of which was used for sequencing and the other half for determining the number of viral copies present. For clinical samples, RNA was extracted from 300 μL of serum, two thirds of which was used for sequencing and the remaining third was used to determine the number of viral copies present. Viral copies were determined using an RT-qPCR assay as described previously using a standard curve to determine the number of viral copies and then calculating the total number of copies sequenced [48].

Since MinION RNA sequencing requires a high amount of input RNA for library preparation (>500 ng), lower viral RNA concentration samples were supplemented with exogenous cellular RNA for sequencing library preparation. Although lower amounts of RNA can be used, adding exogenous mRNA allows for protection of the flow cells, consistency between samples, especially those with low amounts of RNA, and testing of the method for use with clinical samples such as cells or tissues which would contain cellular mRNAs. This exogenous cellular RNA was obtained by extracting total RNA from MARC-145 cells using the Qiagen RNeasy mini kit (Qiagen, Germantown, MD, USA) according to the manufacturer’s protocol with the addition of on-column DNAse digestion. When needed, concentration of RNA was performed using a SpeedVac lab concentrator (Savant, NY, USA). A Qubit 3.0 fluorometer (Life technologies, Carlsbad, CA, USA) and a Nanodrop1000 spectrophotometer (Thermo Scientific, Waltham, MA) were used for quantitative and qualitative assessments. 

### 2.3. MinION Direct RNA Sequencing

Sequencing libraries were generated from 600 ng of extracted viral RNA or a combination of viral RNA and exogenous cellular RNA using the direct RNA sequencing kit (Oxford nanopore Technologies Ltd, Oxford, UK) according to the manufacturer’s protocol [41]. Since the PRRSV genome contains a 3’ poly(A) tail, the standard protocols and DRS adapter provided by Oxford Nanopore were able to be used. The sequencing library was then loaded onto a R9.4.1 SpotON flow cell and sequenced using a MinION Mk I sequencer (Oxford nanopore Technologies Ltd, Oxford, UK) which was connected to a computer and remotely controlled by the MinKNOW software (Oxford nanopore Technologies Ltd, Oxford, UK). The estimated yield was monitored in real-time, samples were sequenced for approximately 6 hours and adjusted for more or less time if needed. 

For evaluation of whole viral genome generation from MinION direct RNA sequencing, two duplicate runs were performed starting with 600 ng PRRSV VR2332 genomic RNA. Sequencing of mixed-strain samples combined 300 ng of VR2332 RNA and 300 ng of strain 1-7-4 or SDEU RNA, or 600 ng VR2332 RNA total as a control. Other samples that contained less than 600 ng of PRRSV RNA, such as clinical samples, were supplemented with exogenous cellular RNA to obtain a total of 600 ng RNA for use in library preparation. 

### 2.4. Evaluation of Sequencing Reads and Consensus Sequences

Basecalling of raw reads was performed using Albacore (Oxford nanopore Technologies Ltd, Oxford, UK) to generate FASTQ files. Total yield, total reads, read quality, and read length from whole genome sequencing were analyzed using NanoPlot [49]. To obtain raw error rates and error patterns, sequencing reads were mapped to the VR2332 reference sequence using minimap2 [50], processed with SAMtools [51] to generate BAM files, and then evaluated by AlignQC [52]. 

A consensus genome was generated using the longest PRRSV read from the sequencing data as a scaffold. The longest PRRSV read was extracted from the FASTQ file using an awk command, all other raw reads were then mapped to this sequence using minimap2 [50], and then the map file was processed using Racon [53]. A comparison of this consensus genome to the reference genome was analyzed by pairwise alignment using Geneious software (version 8.0.5) [54]. Depth of coverage across the consensus genome was analyzed using Qualimap [55]. The average coverage and accuracy across the genome were then evaluated using a window size of 1000 bp and visualized using GraphPad Prism 8 (GraphPad Software, San Diego, CA, USA). 

### 2.5. Evaluation of Analytical Sensitivity

The analytical sensitivity of MinION direct RNA sequencing was analyzed by examining the sequencing yield needed for viral strain detection, as well as the number of viral copies needed to generate detectable viral sequence. The sequencing yield needed for viral strain detection was examined by generating datasets with targeted yields ranging from 3000 to 30,000,000 bases from the two whole genome sequencing runs. Specifically, the text summary of the sequencing file from basecalling was analyzed using R (version 3.4.0) [56] and groups with the desired yields were generated by setting a cutoff at the sequencing time in which the desired yield was reached. Examination of the number of viral copies needed in a sample in order to detect the virus was performed by sequencing viral RNA extracted from cell supernatant samples, spike-in samples, and clinical samples containing different amounts of virus. Because samples with a relatively low number of viral copies yielded low amounts of viral RNA, exogenous cellular RNA was added to achieve efficient library production. Following sequencing of the libraries containing both viral RNA and cellular RNA, the PRRSV sequences needed to be extracted for further analysis. First, a custom PRRSV sequence database containing 951 PRRSV whole genome sequences was generated by downloading all PRRSV whole genome sequences available in GenBank (949 sequences including our VR2332 strain, download date: Nov 2018) with the addition of sequences from our SDEU and 1-7-4 lab strains. Then, the PRRSV reads were able to be identified and obtained by mapping the raw sequencing reads to this custom PRRSV database using minimap2 [50] and extracting the mapped reads using SAMtools [51]. 

Identification of the viral strain present in the sample was examined using basic local alignment search tool (BLAST) with a significance filter of expect value (*E*) < 10^−50^ to examine the PRRSV sequence reads. The PRRSV raw reads were compared to the custom PRRSV database using nucleotide BLAST (BLASTn) and the top match, based on bit score, was regarded as the strain detected in the sample. This detected sequence was then aligned to the known reference genome using Geneious software version R8.0.5 [54] and the percent identity was recorded to show the accuracy of detection. For supernatant and spike-in samples, both the VR2332 whole genome and the ORF5 sequence were known and designated as the reference sequence to compare to the MinION generated sequences. For clinical samples, only the ORF5 sequence was known and was used as the reference sequence for comparison. A consensus genome was generated, if possible, for each dataset or sample using the longest PRRSV read as a scaffold followed by analysis of consensus length and accuracy as described above.

Linear regression analysis was performed to compare PRRSV sequencing reads to viral RNA copies using GraphPad Prism 8 (GraphPad Software, La Jolla, CA, USA). In order to normalize among different sequencing runs with varying total reads, the ratio of PRRSV reads to total reads was used to allow for comparison. The viral RNA copies were determined by RT-qPCR and reported as total viral copies per sequencing run. 

### 2.6. Differentiation of Multiple Viral Isolates in a Single Sample

Samples containing a mixture of two viral isolates, or VR2332 alone as a control, were sequenced as above. In order to identify the yields needed for accurate strain detection and differentiation, datasets with yields from 30,000 to 30,000,000 bases were generated randomly from total reads using fastq-tools (https://homes.cs.washington.edu/~dcjones/fastq-tools/). PRRSV reads were extracted by mapping all reads to the PRRSV database using minimap2 [50]. In order to detect PRRSV strains, PRRSV reads were first BLASTn analyzed to identify the top BLAST hit as determined by bit score (BLAST filter of *E* < 10^−50^ plus alignment identity >80% and length >900 bp). Then, all PRRSV reads were mapped to this top BLAST hit using minimap2 with the “map-ont” preset option [50] and mapped reads were extracted using SAMtools [51]. The unmapped reads were also extracted and were analyzed against the PRRSV database a second time to detect any other strain existing in the same sample. The top BLAST hit was recorded and the mapped and unmapped reads to the second top match were again separated. This was repeated until no PRRSV strain was detected in the extracted unmapped reads. The read length and accuracy were based on the results of the analytical sensitivity experiment, where the detection limit was approximately 900 bp and 80% identity. The top BLAST hits were compared to the targeted known strain (1-7-4, SDEU, or VR2332) and the percent identity was recorded. The percentages of reads matching the detected isolates to total PRRSV reads were also recorded.

The investigation of previous-run contamination was conducted by extracting all reads from the suspected sequencing results that mapped to the reference sequence of the contaminating strain. The “read_id” of the contaminating reads were extracted using SAMtools. As an indication of when during the sequencing run the contaminating read was observed, the “start_time” that matched the “read_id” of the contaminating reads was extracted using R (version 3.4.0) [56]. The number of total contaminating reads over the time course of the sequencing run was analyzed using GraphPad Prism 8 (GraphPad Software, La Jolla, CA, USA). 

### 2.7. Computer Codes and Sequencing Data

The main bioinformatic methods and codes used in this study can be found here: https://github.com/ShaoyuanTan/PRRSVproject.

The sequencing data has been deposited to NCBI Sequence Read Archive (SRA) under accession numbers: SRR10292736 to SRR10292741.

## 3. Results

### 3.1. Evaluation of MinION RNA Sequencing for Generation of Viral Genomes

A high concentration cell culture grown PRRSV VR2332 stock was used for RNA isolation and evaluation of MinION direct RNA whole genome sequencing. PRRSV RNA was extracted using the QIAamp Viral RNA mini kit, which has shown consistently good performance in several studies [57,58]. A total of 600 ng RNA was used for library preparation and sequencing, which was performed in duplicate. Since the whole genome sequencing was under ideal conditions using 600 ng RNA starting material, one-hour of sequencing was sufficient to generate more than enough reads for sequence analysis (Table 1). 

**Figure 1:**
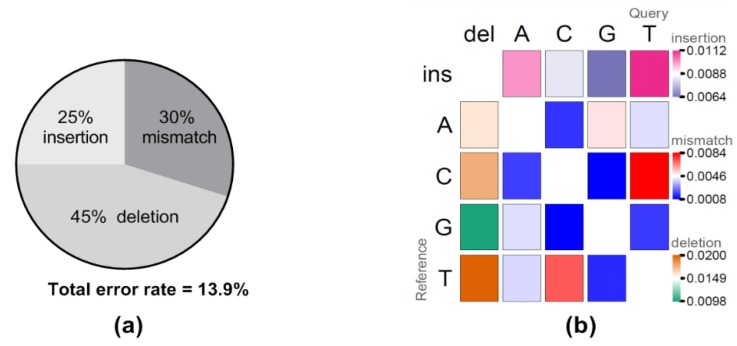
Analysis of direct RNA sequencing errors. To obtain raw error rates and error patterns, raw reads were mapped to the VR2332 reference sequence, followed by evaluation of the mapping. (**a**) The percent of each error type is shown as well as the total error rate. (**b**) The error patterns of insertions (first row with darker pink indicating higher errors), deletions (first column with darker orange indicating higher errors), and mismatches (center matrix with darker red indicating higher error). The U bases in the query sequence were adjusted to T automatically by the minimap program in order to map to the reference sequence which was DNA.

Raw reads from the first hour of sequencing were extracted and evaluated for yield, read quality, read length, raw error rates, and consensus generation (Table 1). Both sequencing runs generated more than 20 megabases (mb) total yield within one-hour of sequencing with the longest raw read over 15,000 bp in length, very close to the full length VR2332 reference sequence (15,182 bp) (Table 1). Interestingly, the majority of the reads were fairly small with only 11–12 reads over 10,000 bp and only 53–73 reads over 7500 bases for the two sequencing runs. Comparing the longest raw read to the VR2332 reference sequence gave an identity of approximately 86.5%, and the sequence accuracy improved to 95.4% after generating a consensus using the longest raw read as a scaffold (Table 1). Further examination of the error rates between the raw reads and the reference sequence identified total error rates at 13.9%, including 6.3% deletion (45% of total error), 4.1% mismatch (30% of total error), and 3.5% insertion (25% of total error) error types (Figure 1a). Of note, error patterns showed that insertion and deletion of U(T) nucleotides, and C/U(T) mismatches were the most frequently observed error patterns (Figure 1b). 

**Figure 2:**
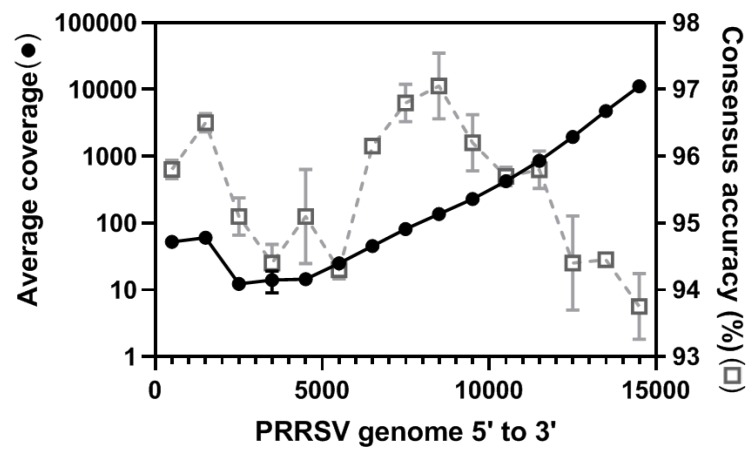
Depth of coverage and consensus accuracy across the porcine reproductive and respiratory syndrome virus (PRRSV) whole genome. Raw reads were mapped to the longest raw read which served as a scaffold to generate a consensus genome. The coverage distribution (left *y*-axis, black closed circles) was evaluated by Qualimap. The consensus accuracy (right *y*-axis, grey open square, dashed line) was generated using a Geneious pair-wise alignment. Both the average coverage and accuracy were evaluated using a window size of 1000 bp and visualized using GraphPad prism software.

The depth of coverage across the PRRSV genome was observed to be extremely uneven with higher coverage on the 3’ end of the genome and gradually decreasing towards the 5’ end, which agrees with what has been observed previously (Figure 2) [44,45]. This is not surprising since the sequence adapter was ligated to the poly(A) tail on the 3’ end and this is where sequencing began. If the RNA was partially degraded or RNA second structure hampered the movement of the RNA through the nanopores, then only the 3’ end would be sequenced, thus resulting in uneven coverage distribution. Despite the uneven coverage, the accuracy across the genome was similar, around 95%, with the middle region of the genome having a higher accuracy (97%) and the 3’ end having the lowest accuracy (93%) (Figure 2). This was surprising since higher coverage can correct random sequencing errors and usually results in higher accuracy, which would suggest the 3’ end would have a much higher accuracy instead of a lower accuracy. Such conflicts imply the existence of technological bias resulting in sequencing errors that cannot be corrected by depth of coverage. A similar observation showing a lower accuracy proximal to the 3’ poly(A) tail has been observed previously due to the DNA adapter, which can partially explain poor accuracy at the 3’ end [41,59]. 

### 3.2. Analytical Sensitivity of MinION Direct RNA Sequencing

#### 3.2.1. Examination of Sequencing Yield Needed for Strain Detection

Analytical sensitivity of MinION direct RNA sequencing was first evaluated by examining sequencing results over a range of sequence yields to determine the lowest sequencing yield at which the PRRS virus could be identified and at which a consensus genome could be generated. A range of sequence yields from 3 kilobases (kb) to 30,000 kb were obtained from the two whole genome sequencing runs above. Total reads were analyzed against a custom PRRSV database using BLASTn and the top match for each sequence yield, even those with only a few reads, was GenBank ID KC469618.1 (15,458 bp). A 99.9% identity was observed between the known sequence of the VR2332 strain used in this experiment (GenBank ID EF536003.1, 15,182 bp) and the top BLAST match, KC469618.1, with an alignment length of 15,183 bp and only 15 base changes, suggesting they are basically the same isolate, especially since PRRSV has a high mutation rate estimated at (4.71–9.8) × 10^−2^/ site/year [20]. 

The length and accuracy of the longest reads, and generation of consensus sequences were further examined at the different sequence yields (Table 2). As sequencing yield increased, the length of the longest reads obtained increased, as did the length of the consensus sequence, reaching a maximal level at a yield of 15,000 kb (Table 2). The accuracy of the longest read at the different yields did not change. However, the accuracy of the consensus sequence increased from about 92% to 95% from 15 kb to 7500 kb input yield, due to the increased depth of coverage (Table 2). Consensus accuracy generated from yields more than 7500 kb was consistently above 95% (Table 2). A nearly full length, 15,101 bp in length (breadth of coverage 99.5%), PRRSV consensus genome sequence with a sequence accuracy of 95.2%, was generated from a sequence yield of 15 mb (Table 2). The minimal sequencing yield required for accurate PRRSV strain detection was found to be 3 kb (~5 reads) (Table 2). A total sequencing yield of 15 mb (~6 × 10^4^ reads) allowed for accurate whole PRRSV genome generation (Table 2).

#### 3.2.2. Determination of Minimal Viral Copy Level needed for Sequencing

The high amounts of viral RNA used for evaluation of MinION sequencing yields above are unrealistic and do not represent amounts of virus that can be found in field samples. Thus, analytical sensitivity was next examined using samples with a more realistic amount of viral copies present. A total of 5 lower concentration cell culture samples, 3 serum samples with known amounts of virus spiked-in, and 6 clinical samples containing varying amounts of virus were sequenced. The total number of viral copies that were used for each MinION sequencing reaction was determined using RT-qPCR, with a range of 3.2 × 10^4^ to 5.9 × 10^9^ viral copies per sequencing reaction in these samples (Table 3). The PRRSV strain was determined by analyzing total raw reads from sequencing against the custom PRRSV database and the top BLAST match was used to identify the viral strain present in the sample (Table 3). MinION sequencing was able to detect PRRSV in spike-in samples containing as low as 3.4 × 10^4^ viral copies and in clinical samples at 3.8 × 10^6^ viral copies (Table 3). The analytical sensitivity difference related to sample type was unexpected, but, in fact, reasonable. One possible reason for this sensitivity difference could be related to viral RNA quality. Viral RNA extracted from cell culture supernatants are produced cleanly in a lab and are quickly stored properly to minimize viral and RNA degradation, thus giving higher quality samples. Clinical samples, on the other hand, are usually obtained on farm and the subsequent handling, shipping, and storage of clinical samples will inevitably increase viral and RNA degradation and decrease sample quality, resulting in lower sequencing yields, while RT-qPCR, which is less sensitive to these conditions, can still detect the presence of the virus [60].

The detection accuracy of the raw PRRSV reads was determined by comparing the top BLAST hit to the known ORF5 sequence and/or whole genome sequence (Table 3). For cell supernatant and spike-in samples, the detection accuracy remains almost the same even as the viral copy number increased from an order of 10^4^ to 10^9^, and the top hits all showed more than a 99% identity to the reference whole genome sequence (Table 3). For clinical samples, at least 3.8 × 10^6^ viral copies were needed in order to detect viral sequence (Table 3). At 3.8 × 10^6^ viral copies the detection accuracy, comparing the top BLAST hit to the known ORF5 sequence, was 94%, increasing to 97% as the number of viral copies increased (Table 3).

PRRSV consensus sequences were obtained from each of the samples, if possible, in order to evaluate the ability of DRS to generate accurate consensus sequence from low viral copy samples (Table 3). MinION sequencing produced a large number of total raw reads, most of which were from the added exogenous cellular RNA necessary for successful library preparation. The desired PRRSV reads were obtained through mapping raw reads against the custom PRRSV database and using those that matched to generate a consensus sequence. A consensus sequence was not able to be obtained for 2 of the samples (spike-in 3.4 × 10^4^ viral copies and clinical 3.8 × 10^6^ viral copies) because of the low number of PRRSV reads present, so the longest PRRSV read was used for accuracy analysis instead of a consensus sequence (Table 3). The accuracy of the consensus sequence (or longest PRRSV read) was determined by comparing it to the known whole genome and/or ORF5 sequence (Table 3). Not surprisingly, there was a general trend that longer and more accurate consensus sequences were generated when more viral copies were sequenced, with slight fluctuations due to variations in sequencer performance (Table 3). Notably, a basically full-length genome with a consensus accuracy of 93.0% was observed in the spike-in sample containing 1.5 × 10^9^ viral copies (Table 3). The other three samples in which more than 10^9^ viral copies were used as the input sample were also able to generate a consensus genome with an accuracy higher than 93%, but were not full-length genomes, perhaps due to the low number of PRRSV reads (and total reads) even though the percent of PRRSV reads per total reads was higher in these samples. Thus, more than 10^9^ viral copies with perhaps 1500 PRRSV reads are recommended if the goal is to obtain a full-length genome sequence, but if identification of the viral strain involved in infection is all that is needed, then clinical serum samples need only have 10^6^–10^7^ viral copies to be successful (Table 3). 

#### 3.2.3. Determination of Sequencing as a Quantitative Method

A comparison between the number of viral copies and the number of viral reads from sequencing was performed to determine if there was a quantitative relationship between input PRRSV RNA amounts and output PRRSV sequencing reads. Of note, the total raw reads varied greatly even though the same amount of total RNA was used for library preparation (Table 3), which was mainly due to the variation of flow cell performance, such as available pores. In order to normalize the comparison, the ratio of PRRSV reads to total reads was calculated and compared to the input viral copies and a strong positive correlation (*r*^2^ = 0.88) was observed. This preliminary result suggests that the knowledge of the number of viral copies in a sample can predict the approximate number of raw reads that will be obtained after sequencing allowing for more successful sequencing results and the number of reads obtained from sequencing can be used to estimate the number of viral copies present in a sample.

### 3.3. Detection of Multiple Viral Isolates Present in a Single Sample

In swine farms, PRRS outbreaks can occur even in herds that are vaccinated, therefore it is necessary to be able to differentiate the presence of infectious field strains from vaccine to aid in outbreak investigation [13,14,16]. To address this issue, we explored the use of MinION DRS for detection of multiple PRRSV strains in the same sample using a stepwise BLAST approach. Samples were created that contained the VR2332 strain (parental strain to the type 2 PRRSV MLV vaccine) to represent vaccine, and either a type 1 PRRSV strain (SDEU, 61.4% similarity with VR2332) or another type 2 PRRSV strain (1-7-4, 82.4% similarity with VR2332). After sequencing, PRRSV reads were extracted from total reads. PRRSV reads were BLAST analyzed against the custom PRRSV database to identify the top match strain and all PRRSV reads that were able to map to this strain were obtained. The unmapped reads were then BLAST analyzed a second time against the custom PRRSV database to identify the top match of these remaining sequences and they were then mapped to this second top match. If unmapped sequences remained, this pipeline was repeated to identify more than 2 PRRSV strains present in the sample. Results showed that even at a total sequence yield of 30 kb (20–26 PRRSV reads), MinION sequencing was able to identify a PRRSV strain with >99.9% identity to the input VR2332 strain (Table 4). The control samples did not identify a second PRRSV sequence present (at any sequence yield) which was promising, since VR2332 was the only virus present. In the mixed virus samples, the second viral strain was not detectable at a total yield of 30 kb. However, at 300 kb or higher yields (245 or more PRRSV reads), the second strain could be identified with an accuracy >99.8% (Table 4). Thus, if enough virus is present from both strains, they could be successfully detected in a single sample. Interestingly, in the VR2332 + 1-7-4 sample, SDEU sequences were also detected, which was not expected since that strain was not present in the sample. Previously, others have observed between-run carryover contamination on the same MinION flowcell [61,62]. Our observation also indicates the carryover contamination from our previous VR2332 + SDEU sample sequencing. This reiterates the need for effective washing of flow cells, as well as good records of what was run on each flow cell previously, especially if flow cells are used for diagnostics. Further investigation into the SDEU carry over contamination showed that SDEU reads were consistently generated during the entire sequencing run, thus contaminating reads could not be minimized by removing the first few minutes of sequencing, they contaminated the entire sequencing run. Although this experiment was designed to differentiate field strains from vaccine strain, it can be applied to the investigation of multiple co-infection strains. Since the identification of the strains present is based on the top BLAST match, any strain with a known genome or similar genome to one in the database could be identified. If no similar strains are present in the database there should be a higher than usual percent of unmapped reads indicating a problem with the BLAST match parameters. The strains examined here were present in equal amounts and had at least an 82.4% identity. Further investigation of strains at different ratios and with higher identity to each other needs to be examined to determine if they would both be able to be distinguished, but with an adjustment of the minimap parameters used to map reads to the top BLAST hit, they should be able to be observed.

From this study we also noticed that the percentage of PRRSV reads that mapped to the first BLAST hit could be used as an indicator for the presence of other PRRSV strains (Table 4). The samples that only contained VR2332 had >98% of PRRSV reads mapping to VR2332, while in the mixed strain samples less than 85% of the PRRSV reads mapped to the first BLAST match, VR2332 (Table 4). 

## 4. Discussion

PRRSV has been a severe threat to the swine industry worldwide ever since it was first described in the late 1980s [63]. Control of PRRSV is difficult, but important for animal welfare and swine production, where the development and implementation of reliable, accurate, and rapid diagnostic methods play a key role. Several methods have been developed and applied to PRRSV diagnosis, which are well described by Ko et al. [64]. Currently, PRRSV diagnostics mainly includes anti-PRRSV antibody detection by serological testing and nucleic acid detection using PCR based assays. Sequencing of PRRSV began in the mid-1990s, to discriminate between strains, which mainly focused on open reading frame 5 (ORF5) or other short regions of interest, but rarely encompassed the complete genome due to technological and monetary limitations [65,66]. PRRSV ORF5 shows extensive genetic diversity and has been used for providing insight into PRRSV epidemiology, however it is only 5% of the whole genome, thus 95% of the genomic information remains for prediction of genetic variation. Whole genome sequencing is greatly needed to provide a more complete picture of the virus [67,68], which is now gradually becoming more feasible with the rapid development and innovation of new sequencing technologies [69,70]. Oxford Nanopore direct RNA sequencing (DRS) is revolutionary for sequencing RNA viral genomes, since it can sequence the RNA directly, allowing for detection of methylation sites and decreasing bias inherent in reverse transcription and PCR amplification of samples prior to sequencing, and it can generate long reads, allowing for the elucidation of recombination events [71]. 

This study was planned and performed to assess the feasibility of Oxford Nanopore MinION DRS in clinical PRRSV diagnostics to identify the viral strains involved in infection. The key interests addressed in this study included whether sequencing can detect PRRSV strains to identify an outbreak as occurring due to the introduction of a new strain or recirculation of a previous outbreak, whether sequencing can generate whole genome information to aid in further understanding of PRRSV epidemiology, and whether sequencing can detect and differentiate multiple strains in a single sample to investigate outbreaks that occur in vaccinated herds or co-infection of multiple field-strains. Previously, PRRSV whole genomes have been generated using Sanger and Illumina sequencing technologies [10,47,72]. While both sequencing technologies can generate a whole PRRSV genome with more than 99.9% accuracy, the raw reads produced are usually less than 1500 bp. As a result, in order to generate a PRRSV whole genome, multiple primer sets and multiple individual sequencing reactions are needed for Sanger sequencing which is labor and time consuming; or for Illumina, computing resource intensive genome assembly is needed which requires time and knowledge to perform effectively. Oxford Nanopore MinION sequencing, on the other hand, can generate ultra-long raw reads which are in theory only limited by input fragment length [73]. This feature is beneficial, since it saves time and effort when generating a whole genome sequence. In this study, we successfully generated PRRSV raw reads up to the length of the entire genome (15 kb) with an approximate 86% identity to the known input genome sequence. A bioinformatics approach was developed that used the longest raw read as a scaffold to effectively generate a consensus sequence, improving the accuracy to 96% identity of the input genome. 

Sequencing can be incorporated as a supportive tool for PCR to aid in diagnostic strain level PRRSV detection. It has been reported that both Sanger and Illumina sequencing can accurately detect PRRSV strains present in a sample, but both require transcription of RNA into cDNA followed by PCR amplification prior to sequencing [10,72]. Differing from this, MinION technology directly sequences RNA strands for detection of PRRSV strains. This feature is beneficial since no reverse transcription or PCR are needed thus eliminating biases that those introduce and saving time since extra steps need not be performed, which allows for same day disease investigation. Moreover, direct RNA sequencing allows for the detection of nucleotide analogs which have been correlated with numerous diseases [74]. Most importantly, the MinION sequencer is cost-effective and easily accessible, without the investment of expensive sequencing and bioinformatics infrastructure. Despite the low raw read accuracy of direct RNA sequencing (~86%), which is the main concern with this technology, PRRSV strains were identified with 99.9% accuracy using as few as 5 raw reads (3 kb total yield). This accurate strain-level detection, even though the sequence accuracy is low, allows for guidance on determining effective control methods due to the precise detection of the circulating strains on a farm. 

Now knowing the potential of DRS for strain level detection of pathogens as determined through this study as well as others [75], we next investigated the analytical sensitivity of PRRSV detection to determine its usefulness for obtaining reliable sequencing results. Previous research examining analytical sensitivity of next-generation sequencing has reported sensitivities that are similar or less sensitive than RT-qPCR [28,76], and the third-generation Oxford Nanopore DRS has previously shown a sensitivity of 1.89 × 10^7^ viral copies in an influenza virus study [44]. Our results indicated that samples with a minimum of 10^4^ to 10^6^ viral copies, depending on the sample type, can be successfully sequenced to accurately identify strains after about 6 hours of sequencing. Although DRS is not as sensitive as PCR for use as a diagnostic tool identifying viral presence [77,78], it can be used for further investigation of the strain causing an outbreak, either directly from high viral load samples or following amplification of virus in cell culture. Additionally, a very strong correlation was observed between the number of viral reads generated through sequencing and the starting number of viral copies, indicating sequencing reads can be predicted by viral copies in a sample and vice versa, which has been confirmed by other studies as well [28]. Interestingly, the observation that the sensitivity of sequencing was higher from cell culture virus spiked into serum as opposed to clinical serum samples suggests that sample handling or perhaps the quality of the sample was an important factor for sequencing sensitivity [79], thus emphasizing the importance of careful handling, transporting, and storing of clinical samples to protect the viral RNA from degradation [80,81]. This also suggests that on-site sequencing of samples as opposed to a centralized diagnostic system may allow for higher sensitivity of detection due to the ability to immediately process samples after sampling.

In addition to a single strain infection, clinical situations have been shown to be more complicated, sometimes involving infection with multiple strains simultaneously, such as co-infection of multiple field strains or co-existence of field strain(s) with vaccine strain [15,82]. This not only poses challenges to disease diagnosis but also increases the chance of PRRSV recombination, which is considered to be one of the most important mechanisms in PRRSV evolution [10,83]. In order to address this issue, Oxford Nanopore DRS was evaluated to determine if it could be used to discriminate co-infection by two PRRSV strains from different genotypes (61.4% similarity) as well as from the same genotype (82.4% similarity) in a single sample. In fact, the strains were easily differentiated, and the same method could be used to identify more than 2 strains present in a single sample.

This study begins the process of developing rapid and high-resolution PRRSV diagnostics for use in clinical situations where genomic data is urgently needed. This includes situations of potential infection, outbreak investigation, vaccine design guidance, and producer desires for more specific information. The PRRSV RNA genetic material presents the same technical demands for extraction, processing, and sequencing as do influenza virus, coronaviruses, picornaviruses, rotaviruses, and many foreign animal disease viruses for which rapid pathogen identification and discrimination can be critically important. Knowledge gained from PRRSV in this study can be immediately translatable to aid in rapid diagnostic detection and strain-specific identification of an entire class of important swine pathogens. In fact, MinION sequencing technology might end up being a useful and affordable diagnostic tool for swine veterinary medicine in general. This technology can provide a complete readout of RNA viruses and RNAs from the host or other pathogens present in a sample without the need for pre-existing knowledge of what might be present [84].

The current evaluation of this sequencing technology indicates that it can be used successfully along with qPCR for diagnosis of a pathogen, whole genome generation, strain-level pathogen detection and differentiation. As the DRS technology continues to develop and RNA isolations are optimized for use outside of a research laboratory, these methods can be further refined and optimized using updated materials and protocols. The future goal is to realize on-site infectious disease investigation using the Oxford Nanopore MinION portable sequencer to allow for quicker diagnosis and facilitation of more rapid decision-making, an important consideration in an industry in which delays in moving animals due to unknown health status can disrupt flow patterns and schedules, or cause disease outbreaks with great economic losses. 

## Figures and Tables

**Table 1:** Assessment of raw reads from direct RNA sequencing.

Sequencing Statistic	Run #1	Run #2
Available pores (group 1)	474	495
Sequencing time	1 hour	1 hour
Total pass bases	20,351,741	27,167,775
Total pass reads	14,963	23,547
Mean read length (bp)	1360	1154
Mean read quality	8.2	8.5
Mappable reads/percentage	13,284/88.8%	19,549/83.0%
Longest read (bp)/accuracy	15,026/86.3%	15,060/86.7%
Consensus length (bp)/accuracy	15,140/95.5%	15,055/95.3%

**Table 2:** Detection power of MinION direct RNA sequencing at different sequence yields ^a^.

Sequencing Yield (kb)	Number of Total Reads	Longest Read		Consensus Sequence		Coverage	
		Length (bp)	Accuracy	Length (bp)	Accuracy	Breadth ^b^	Depth ^c^
3	5	1606	84.2%	-	-	-	-
15	109	1899	88.1%	1861	92.10%	12.3%	1
75	443	4036	84.1%	4081	91.95%	26.9%	5
150	790	4496	83.4%	4548	92.45%	30.0%	10
750	3500	7533	84.9%	7609	92.55%	50.1%	49
1500	6857	8382	85.8%	8435	94.20%	55.6%	99
7500	32,571	12,990	86.6%	12,988	95.20%	85.5%	494
15,000	64,860	15,043	86.7%	15,101	95.20%	99.5%	988
30,000	127,411	15,081	86.0%	15,171	95.50%	99.9%	1976

^a^ average of two sequence runs; ^b^ consensus genome length (bp)/reference genome length (bp); ^c^ sequencing yield (bp)/reference genome size (bp).

**Table 3:** Analytical sensitivity of direct PRRSV RNA sequencing.

Sample Type	Viral Copies/ Reaction	# of Total Reads	Top BLAST Match	Identity to ORF5/Whole Genome %	# of PRRSV Reads	Consensus/Longest Read	
						Length (bp)	Accuracy %
Cell supernatant	5.9 × 10^9^	19,198	KC469618.1	100.0/99.9	1247	8282	94.5
	2.0 × 10^9^	23,068	KC469618.1	100.0/99.9	949	7167	93.8
	1.7 × 10^9^	83,192	KC469618.1	100.0/99.9	831	9187	93.2
	6.8 × 10^8^	116,698	KC469618.1	100.0/99.9	699	5975	93.1
	3.7 × 10^8^	118,879	KC469618.1	100.0/99.9	422	6028	93.7
Spike-in	1.5 × 10^9^	322,778	KC469618.1	100.0/99.9	1589	15,021	93.0
	9.4 × 10^6^	300,143	KC469618.1	100.0/99.9	45	3743	90.5
	3.4 × 10^4^	161,569	CS484777.1	99.0/99.4	3	905 *	82.1
Clinical	1.4 × 10^8^	77,468	MF327000.1	96.8/-	42	1984	90.5
	2.4 × 10^7^	266,120	KX192112.1	97.0/-	16	2431	88.4
	3.8 × 10^6^	286,680	KT581982.1	94.4/-	6	940 *	83.7
	2.3 × 10^5^	201,887	ND	-	0	-	-
	6.5 × 10^4^	240,944	ND	-	0	-	-
	3.2 × 10^4^	307,822	ND	-	0	-	-

ND: not detected; * Longest raw read was used.

**Table 4:** Mapping status of direct RNA sequencing on samples containing multiple viral strains.

Groups	Total Yield (kb)	# of Total Reads	# of PRRSV Reads	PRRSV Reads /Total Reads	First Match			Second Match			Third Match		
					Top BLAST Match (Identity %)	# of Matching Reads	% of PRRSV Reads	Top BLAST Match (identity %)	# of Matching Reads	% of PRRSV Reads	Top BLAST Match (identity %)	# of Matching Reads	% of PRRSV Reads
Control	30	25	20	80%	KC469618.1 (99.9)	20	100%	ND					
	300	245	210	86%	KC469618.1 (99.9)	208	99%	ND					
	3000	2451	2079	85%	KC469618.1 (99.9)	2044	98%	ND					
	30,000	24,512	20,819	85%	KC469618.1 (99.9)	20,472	98%	ND					
VR2332 + SDEU mixed sample	30	38	23	61%	KC469618.1 (99.9)	19	83%	ND					
	300	375	234	62%	KC469618.1 (99.9)	194	83%	CS421743.1 (99.8)	35	15%	ND		
	3000	3748	2281	61%	KC469618.1 (99.9)	1722	75%	SDEU (100.0)	514	23%	ND		
	30,000	37,478	23,004	61%	KC469618.1 (99.9)	17,610	77%	SDEU (100.0)	4879	21%	ND		
VR2332 + 1-7-4 mixed sample	30	34	26	76%	JA894280.1 (100.0)	18	69%	ND					
	300	335	272	81%	KC469618.1 (99.9)	224	82%	1-7-4 (100.0)	38	14%	ND		
	3000	3351	2699	81%	KC469618.1 (99.9)	2287	85%	SDEU (100.0)	14	1%	1-7-4 (100.0)	348	13%
	30,000	33,512	26,917	80%	KC469618.1 (99.9)	22,668	84%	SDEU (100.0)	136	1%	1-7-4 (100.0)	3633	13%

ND: not detected.
